# Safety and efficacy of S1 monotherapy or combined with nab-paclitaxel in advanced elderly pancreatic cancer patients: A meta-analysis

**DOI:** 10.1097/MD.0000000000026342

**Published:** 2021-06-25

**Authors:** Yunlong Chen, Jiangning Gu, Menghong Yin, Chenqi Wang, Dan Chen, Lili Yang, Xiang Chen, Zhikun Lin, Jian Du, Shimeng Cui, Chi Ma, Haifeng Luo

**Affiliations:** a Department of Hepatobiliary Surgery, the First Affiliated Hospital of Dalian Medical University; b Dalian Central Hospital; c Department of Pathology, the First Affiliated Hospital of Dalian Medical University, Dalian, China.

**Keywords:** chemotherapy, elderly patients, meta-analysis, nab-paclitaxel, PDAC, S1

## Abstract

**Objective:**

To evaluate the therapeutic efficacy and safety of S1 monotherapy or combination with nab-paclitaxel for the treatment of elderly patients with metastatic or locally advanced pancreatic adenocarcinoma.

**Method:**

PubMed, Embase, Cochrane Central Library, China Biology Medicine, and China National Knowledge Infrastructure databases were searched without time limits according to the inclusion criteria. RevMan (Version 5.3) software was used for data extraction and meta-analysis. Objective response rate (ORR) and disease control rate (DCR) were used to evaluate therapeutic effects while side effects including leukopenia, thrombocytopenia, neurotoxicity, vomit, and alopecia were extracted for evaluation. There was no need for ethical review in this study because no ethical experiments were conducted and all data used were public data. All relevant data are within the paper and its Supporting Information files.

**Results:**

Four retrospective studies comprising 308 elderly patients with metastatic or locally advanced pancreatic adenocarcinoma were included in the analysis. One hundred fifty-one patients underwent S1 monotherapy and 157 received S1 combined nab-paclitaxel. Meta-analysis indicated that compared with S1 monotherapy, S1 combined with nab-paclitaxel had higher ORR (OR 2.25, 95% CI: 1.42–3.55; *P* = .0005) and DCR (OR 2.94, 95% CI: 1.55–5.58; *P* = .0009). The adverse reaction of leukopenia was higher in the combined therapy group (OR 1.85, 95% CI: 1.09–3.13, *P* = .02), but no significant difference was found in thrombocytopenia, neurotoxicity, vomiting, and alopecia between the 2 groups (*P* > .05).

**Conclusion:**

Nab-paclitaxel plus S1 was more efficient in terms of ORR and DCR than S1 monotherapy in elderly pancreatic ductal adenocarcinoma patients while the side effect was controllable with a higher probability of leukopenia. Thus, combined nab-paclitaxel and S1 could be safely used in elderly patients.

## 1 Introduction

Pancreatic cancer is a highly malignant gastrointestinal tumor with hidden symptoms, rapid progression, and poor prognosis. The lack of early specific symptoms suggests that most of patients with pancreatic ductal adenocarcinoma patients (PDAC) are diagnosed at a late stage; thus, only less than 20% of patients could undergo radical surgery, and even after this, the cancer recurrence rate is still high.

Based on this, the latest version of the NCCN guidance clarifies the role of chemotherapy in the comprehensive treatment of PDAC, including resectable, borderline resectable, locally advanced and metastatic PDAC. However, although combined chemotherapy protocols, such as FOLFIRINOX and AG, could prolong the overall survival compared with gemcitabine monotherapy, the incidence rate of adverse reactions also increases.^[1,2]^ Due to the racial differences, the response of different chemotherapeutic regimens is not always the same between eastern and western populations.^[3]^ For example, the FOLFIRINOX was the first recommended chemotherapy in American and European countries, but most of the Asian patients could not bear their side effects and lead to temporary cessation of treatment. S1, a new oral form of fluorouracil derivative, was developed by Japanese scholars and validated to have a longer overall survival (OS) and progression-free survival (PFS) compared with monotherapy in Asian patients with PDAC than in Western patients.^[4]^

Similar to other types of cancer, the incidence of PDAC increases with age, and is expected to reach its peak in 60 years, while by the year of 2030, it is estimated that nearly 70% of PDAC will be diagnosed in the elderly population.^[5]^ The WHO defines elderly patients as those over 65 years old, and China defines elderly patients as those over 60 years old. In this paper, people over 60 years old are defined as the elderly. This situation also makes a dilemma in clinical practice in elderly PDAC patients, since according to experience this group was always not recommended to intensive treatment, partly out of the fear that these medications may be a potential stronger “striker” instead of tumor itself. Thus, the treatment of elderly PDAC patients still lacks evidence of high quality. This study aimed to evaluate the efficacy of S1 monotherapy and combination with nab-paclitaxel in elderly patients with advanced PDAC by meta-analysis and provide some reference for treatment in the near future.

## 2 Patients and methods

### 2.1 Search strategy

PubMed, Embase, Cochrane libraries, China Biology Medicine, and China National Knowledge Infrastructure were searched without a time limit. The language of this article is limited to Chinese and English. The appropriate studies were searched using the following MeSH terms: pancreatic neoplasms AND nab-paclitaxel AND S1.

### 2.2 Inclusion and exclusion criteria

Inclusion criteria: Types of study: randomized controlled and case-control studies were included in this meta-analysis. Patients: elderly locally advanced and metastatic PADC patients (≥ 60 years old) who did not receive other anti-cancer therapy before being included in the study; there were no other tumors except pancreatic cancer; blood RT, liver, and renal function were basically normal with good tolerance (ECOG≤2). Interventions: S1 monotherapy and S1 combined with nab-paclitaxel. Outcome indicators: the therapeutic effect indexes include objective response rate (ORR) and disease control rate (DCR), OS, and PFS. The main adverse reactions were leukopenia, thrombocytopenia, neurotoxicity, vomiting, and alopecia. Exclusion Studies of low quality (NOS) less than or equal to 5. Studies without a control group, such as case reports or single-arm studies.

### 2.3 Data extraction

Data extraction and quality assessment were independently performed by the 2 reviewers. Any disagreements between the reviewers were discussed with a third reviewer to achieve a consensus. The following information was collected: publication time, name of the first author, number of cases, sex, age, treatment plan, ORR, DCR, OS, PFS, and adverse reactions in different studies.

### 2.4 Evaluation criterion

According to the Response Evaluation Criteria in Solid Tumors or the Response Evaluation Criteria in Solid Tumors of the WHO^[6]^ to evaluate treatment efficacy. It is divided into complete remission (CR), partial remission (PR), stable disease (SD), and disease progression. The effective rate was calculated by CR + PR, and the disease control rate was calculated by CR + PR + SD. The occurrence of adverse reactions was evaluated according to the National Cancer Institute Common Terminology Standard for Adverse Reactions.^[7]^

### 2.5 Statistical analysis

Data was processed with Revman5.3, the differences between the 2 groups of categorical variables were compared by odds ratio (OR) and 95% confidence interval (CI). The differences of continuous variables were compared with each other by corresponding weighted mean difference and 95% CI. The I^2^ test and Egger test were used to evaluate the heterogeneity and bias among the studies, respectively. Studies with satisfactory homogeneity (*P* > .1, I^2^ < 50%) were analyzed using the fixed-effect model. The source of heterogeneity was further analyzed when the heterogeneity was significant (*P*≤0.1, I^2^≥50%) and a random effect model was applied if there was no significant heterogeneity.

## 3 Results

### 3.1 Literature search and selection of the studies

**Figure 1:**
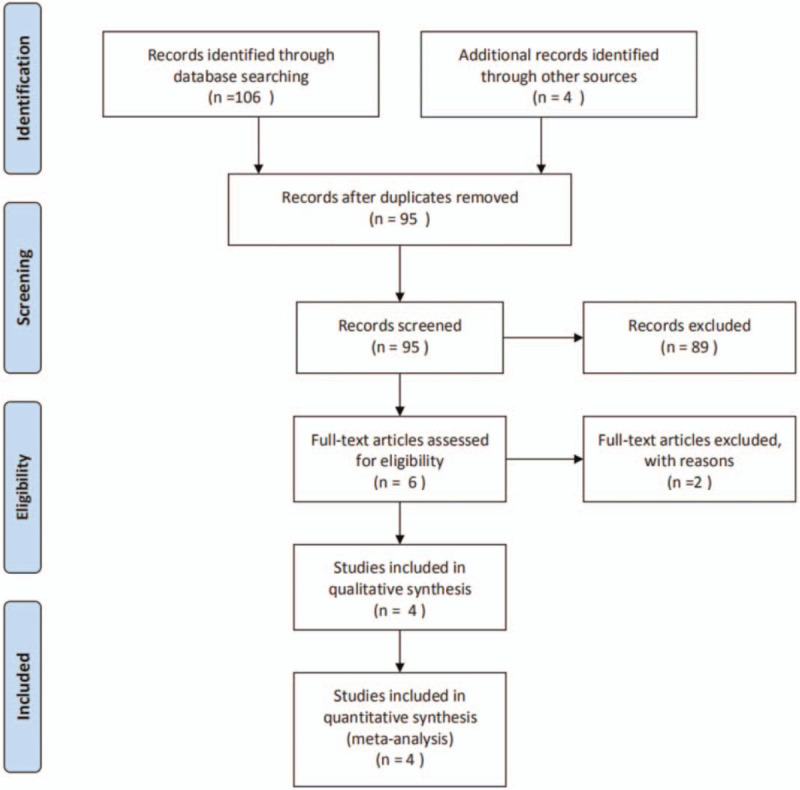
Flow diagram of the study selection.

A total of 110 papers were retrieved, after excluding case reports, duplicated reports, single-arm studies, and studies without sufficient information or with different chemotherapy regimens, we finally selected 4 retrospective studies.^[8–11]^ The selection flowchart is presented in Figure 1. A total of 308 elderly patients with pancreatic ductal adenocarcinoma were included in the analysis. The basic characteristics and quality evaluation results of the included studies are presented in Table 1.

**Table 1:** The basic characteristics and quality evaluation of the included documents.

Author	Time	Number of cases	Treatment plan	Gender (male/female)	Median age (years)	Quality evaluation (points)
Qianqian Han et al^[8]^	2018	40	Nab-P + S-1	27/13	68.4 ± 7.3	8
		46	S-1	29/17	68.7 ± 7.5	
Sirui Li et al^[9]^	2019	30	Nab-P + S-1	18/12	67.21 ± 2.48	6
		30	S-1	17/13	67.46 ± 2.39	
Guang Li^[10]^	2019	47	Nab-P + S-1	26/21	68.74 ± 6.53	7
		47	S-1	25/22	68.76 ± 6.54	
Shiwei Han et al^[11]^	2016	34	Nab-P + S-1	19/15	65.32 ± 7.41	8
		34	S-1	20/14	64.87 ± 7.63	

### 3.2 Results of meta-analysis

#### 3.2.1 Treatment effect

**Figure 2:**
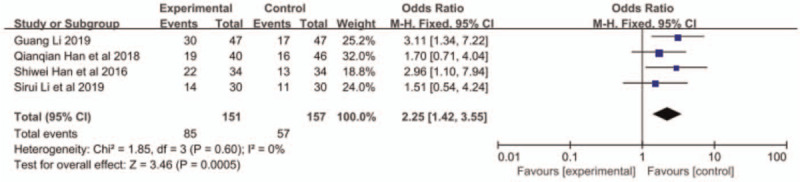
The forest plots of ORR between S-1 monotherapy and S-1 combined with nab-paclitaxel in the treatment of elderly patients with advanced pancreatic cancer. ORR = objective response rate.

**Figure 3:**
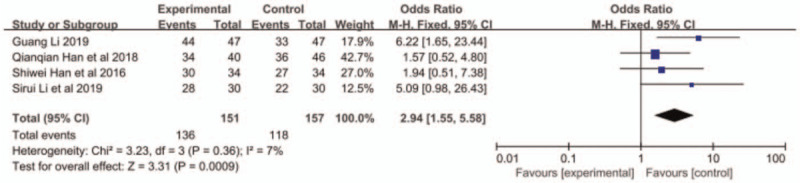
The forest plots of DCR between S-1 monotherapy and S-1 combined with nabpaclitaxel in the treatment of elderly patients with advanced pancreatic cancer. DCR = disease control rate.

All the 4 papers reported ORR and DCR, and small heterogeneity existed among the research results (ORR: *P* = .60, I^2^ = 0; DCR: *P* = .36, I^2^ = 0%). Therefore, the fixed-effects model was applied for further meta-analysis. The results indicated that compared with S1 monotherapy, nab-paclitaxel combined with S1 had a better response rate with higher ORR (OR = 2.25, 95% CI: 0.85–2.16, *P* = .0005, Fig. 2) and DCR (OR = 2.94, 95% CI: 1.55–5.58, *P* = .009, Fig. 3).

#### 3.2.2 Adverse reactions of different modalities

**Figure 4:**
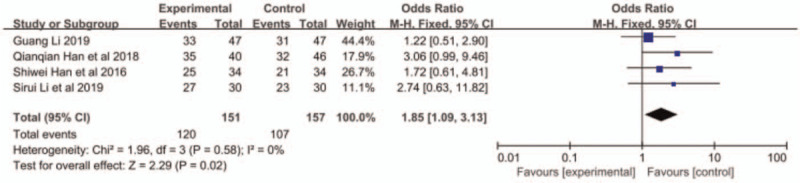
The forest plots of leukopenia between S-1 monotherapy and S-1 combined with nab-paclitaxel in the treatment of elderly patients with advanced pancreatic cancer.

**Figure 5:**
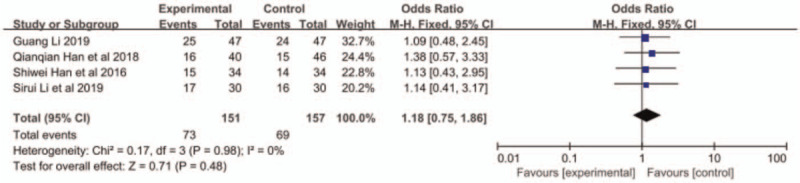
The forest plots of nausea and vomiting between S-1 monotherapy and S-1 combined with nab-paclitaxel in the treatment of elderly patients with advanced pancreatic cancer.

**Figure 6:**
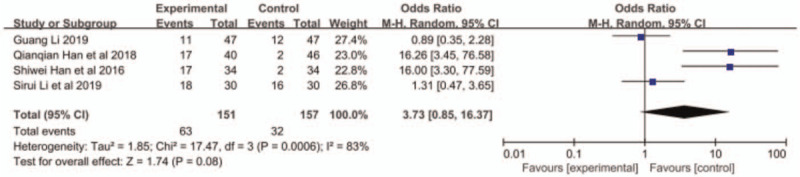
The forest plots of neurotoxicity between S-1 monotherapy and S-1 combined with nab-paclitaxel in the treatment of elderly patients with advanced pancreatic cancer.

**Figure 7:**
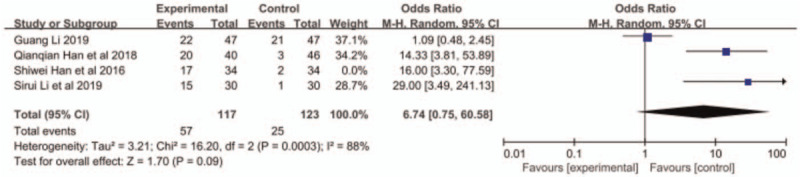
The forest plots of alopecia between S-1 monotherapy and S-1 combined with nab-paclitaxel in the treatment of elderly patients with advanced pancreatic cancer.

**Figure 8:**
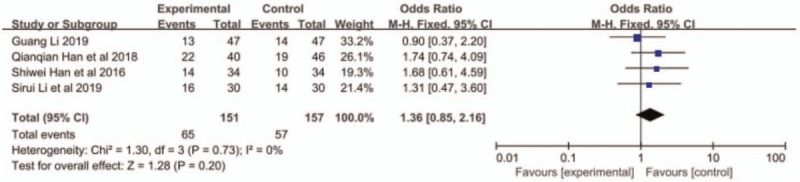
The forest plots of thrombocytopenia between S-1 monotherapy and S-1 combined with nab-paclitaxel in the treatment of elderly patients with advanced pancreatic cancer.

Adverse reactions included leukopenia, thrombocytopenia, neurotoxicity, vomiting, and alopecia. Han et al^[11]^ did not report the occurrence of alopecia. The other 3 articles reported the aforementioned adverse reactions. The results of meta-analysis showed that the incidence of leukopenia in the combined group was higher than that in the S-1 monotherapy group (OR = 1.85, 95% CI: 1.09–3.13, *P* < .05, Fig. 4), but no significant difference was found in other reactions between the 2 groups (Figs. 5–8: nausea and vomiting (OR = 1.18, 95% CI: 0.75–1.56, *P* = .48, Fig. 5), neurotoxicity (OR = 3.73, 95% CI: 0.85–16.37, *P* = .08, Fig. 6), alopecia (OR = 6.74, 95% CI: 0.74–60.58, *P* = .09, Fig. 7), and thrombocytopenia (OR = 1.36, 95% CI: 1.42–3.55, *P* = .20, Fig. 8).

### 3.3 Sensitivity analysis

Each study was excluded separately to verify the reliability of the conclusions. The results showed that the result of ORR (effective rate) and DCR (disease control rate) of the 2 treatment plans did not change significantly. When studies by Han et al^[8]^ and Li et al^[9]^ were excluded, the incidence rate of leukopenia was no longer significant, while the other adverse reactions remained unchanged. This may be attributed to the small size of the included studies.

### 3.4 Bias risk

Egger test was used to observe publication bias and is presented in Table 2. No significant publication bias was found in this meta-analysis, but the possibility of existence could not be completely ruled out. There are only a few published controlled studies associated with chemotherapy in elderly patients with PDAC.

**Table 2:** The results of Egger test.

	Number of studies = 4 root				MSE = 1.747	
Std_Eff	Coef.	Std. err.	*t*	*P*>|t|	[95% conf. interval]	
Slopebias	2.112411	4.850975	0.44	.706	−18.75965	22.98447
−3.619901	10.31241	−0.35	0.759	−47.9906	40.7508	

Test of H0: no small-study effects *P* = .759.

## 4 Discussion

In the last 5 years, the treatment of pancreatic cancer has been from simple surgical treatment to comprehensive treatment, including chemotherapy, targeted treatment, and progressive immunotherapy;^[12]^ among these, the position of chemotherapy in comprehensive treatment of PDAC has been gradually clarified, which could prolong the overall survival. Although the regimen has been studied in depth, the appropriate group for specific regimens has not been fully explicated due to tumor heterogeneity and racial differences. It cannot be denied that cancer itself is a kind of age-related aliment; thus, a considerable proportion of the patients are elderly patients whose general condition is relatively poor, which makes treatment decisions more difficult because they are most likely unable to tolerate intensive chemotherapy. Clinicians are more inclined to prescribed single chemotherapy than combined regimens because of the “poor condition”; however, there is no high-grade evidence to support this idea. In terms of monotherapy, gemcitabine was previously regarded as the standard drug. Recently, S1, a new type of orally administered fluorouracil derivative, was validated as a more effective chemotherapeutics with longer overall survival, especially in the Asian group, and thus, it has become a popular drug in clinical practice.^[13]^ Conjugated with albumin, nab-paclitaxel has a better effect due to its excellent specificity of membrane permeability and thus leads to a higher drug concentration in stroma-rich PDAC tumors;^[14]^ thus, it has been widely recommended in (neo)adjuvant chemotherapy.

The anti-tumor efficacy of nab-paclitaxel combined with S1 has been investigated in several studies. Shi's^[15]^ single-arm, phase II study indicated that the combined therapy for advanced PDAC had an encouraging effect with OS 9.4 (95% CI 8.0–10.8) months, PFS 5.6 (95% CI 4.6–6.6) months, ORR 50% and DCR 71.4%, respectively, while the side effect was manageable. Similar to this result, Zhang's^[16]^ study also indicated the satisfactory effect of combined therapy with ORR (53.1%) and DCR (87.5%), while the OS and PFS were 13.6 (range 8.7–18.5) and 6.2 (range, 4.4–8) months, respectively. Hu and Sun^[17]^ also presented their single-arm results indicating that combined nab-paclitaxel and S1 had a promising anti-tumor effect with an ORR 51.9%, median PFS of 5.7 [95% CI 5.010–6.292] months, median OS 11.9 [95% CI 9.731–13.990] months, respectively, while the toxicities were well tolerated. The above investigations confirmed its efficacy and safety in PDAC patients; however, all of them were single-arm trials and the age was relatively younger, with a median age ranging from 53 to 59 years. Although the above study was not aimed at elderly patients with advanced pancreatic cancer, the results (ORR, DCR, OS, and PFS) are similar to the results of the experimental group included in the study (Han et al^[8]^ and Li et al^[10]^). This implies that nab-paclitaxel combined with S1 in the treatment of elderly patients with advanced pancreatic cancer may have an effect similar to that of young patients. The evidence of the effect of nab-paclitaxel plus S1 compared with S1 monotherapy, especially in elderly patients, still needs to be further explained.

This meta-analysis included the four 2-arm studies targeting elderly patients with advanced PDAC receiving nab-paclitaxel with S1 combined therapy with S1 monotherapy as a control. The median age of the 4 studies was > 65 years old. The effectiveness evaluation was not unexpected; the ORR and DCR were significantly higher in the combined group rather than S1 monotherapy group. The pity of the study is that the OS was not meta-analyzed in this study because only 2 articles recorded the survival time. Han's^[8]^ and Li's^[10]^ study indicated that the OS in combined group was higher than S1 monotherapy group with 9.5 versus 8.2 months and 10.83 versus 7.41 months, respectively. In terms of adverse reactions, except leukopenia was a slightly higher in the combined treatment group, other factors including thrombocytopenia, neurotoxicity, nausea, vomiting, and alopecia were not significantly different. However, sensitivity analysis showed that the difference would no longer exist when 2 studies were excluded, which may be attributed to the sample size to some extent. Thus, the results of the meta-analysis at least validated its safety with higher ORR and DCR in elderly patients.

This study has certain limitations. Except for the lack of OS meta-analyzed mentioned before, the small sample size also had a negative influence on the final result because, on the one hand, studies associated with S1 were mainly carried out in Asian countries; on the other hand, no results of prospective randomized controlled studies have been presented thus far; thus, only retrospective case-control studies were included. Therefore, it is still necessary to conduct multicenter randomized controlled trials with larger sample sizes for further validation.

In conclusion, combined nab-paclitaxel and S1 were more effective than S1 monotherapy in terms of ORR and DCR in elderly patients with advanced PDAC and manageable adverse reactions. This regimen can be safely applied in clinical practice. However, multicenter randomized controlled studies are needed for further verification.

## Acknowledgments

The authors thank Prof Wei Huang for his organization of a biomedical database at Dalian Medical University and Mr Yushan Wei for statistical support. This work was mainly funded by the National Nature Science Foundation of China (No. 81902382) through the Jiangning Gu and Outstanding Doctor Training Program through Haifeng Luo.

## Author contributions

**Conceptualization:** Yunlong Chen, Jiangning Gu, Zhikun Lin, Shimeng Cui, Haifeng Luo.

**Data curation:** Yunlong Chen, Jiangning Gu, Chenqi Wang, Dan Chen, Lili Yang, Xiang Chen, Jian Du, Shimeng Cui, Chi Ma, Haifeng Luo.

**Formal analysis:** Yunlong Chen, Jiangning Gu, Dan Chen, Haifeng Luo.

**Funding acquisition:** Jiangning Gu, Haifeng Luo.

**Investigation:** Yunlong Chen, Chenqi Wang, Zhikun Lin, Chi Ma, Haifeng Luo.

**Methodology:** Yunlong Chen, Haifeng Luo.

**Project administration:** Yunlong Chen, Haifeng Luo.

**Resources:** Yunlong Chen, Menghong Yin, Dan Chen, Zhikun Lin, Chi Ma, Haifeng Luo.

**Software:** Yunlong Chen, Menghong Yin, Chenqi Wang, Xiang Chen, Jian Du, Haifeng Luo.

**Supervision:** Yunlong Chen, Jiangning Gu, Haifeng Luo.

**Validation:** Yunlong Chen, Haifeng Luo.

**Visualization:** Yunlong Chen, Haifeng Luo.

**Writing – original draft:** Yunlong Chen, Haifeng Luo.

**Writing – review & editing:** Yunlong Chen, Jiangning Gu, Haifeng Luo.
